# A Two-Level Preconditioner for the CASSCF Linear-Response Equations

**DOI:** 10.1021/acs.jpca.5c04385

**Published:** 2025-08-21

**Authors:** Benjamin Helmich-Paris

**Affiliations:** 28314 Max-Planck-Institut für Kohlenforschung, Kaiser-Wilhelm-Platz 1, D-45470 Mülheim an der Ruhr, Germany

## Abstract

We present an efficient two-level strategy to accelerate the solution of the CASSCF linear-response eigenvalue problem using a customized Davidson algorithm. By identifying a subset of important response-vector components—the so-called P space—we compute and diagonalize full Hessian and metric matrix elements while treating the remaining Q-space components with a diagonal approximation. This approach decouples the orbital and configuration responses, enabling independent preconditioning of each component. Computational cost is further reduced through the resolution-of-the-identity approximation. We demonstrate significant performance gains across a diverse set of molecules, achieving speedups of up to 2.05 compared to the standard diagonal preconditioning. The largest efficiency gains are observed for MCTDA calculations involving many excited states and relatively small response-vector lengths. The two-level strategy is available in ORCA 6.1 and paves the way for extensions to dynamic polarizabilities, which require solving large-scale linear equations, as well as to time-dependent density functional theory and CI singles.

## Graphical abstract

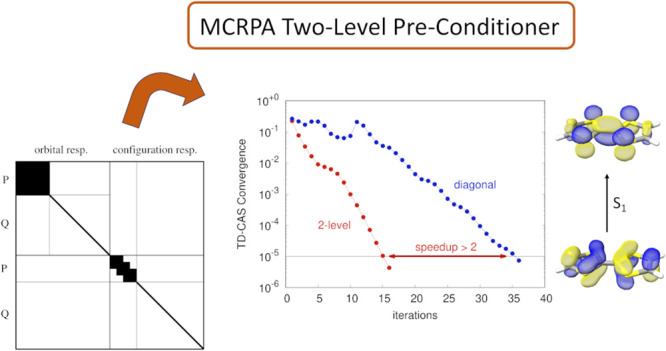

## 1 Introduction

Simulating molecules with complex electronic structures remains a significant challenge in quantum chemistry. This is especially true for systems with open-shell configurations as they occur in transition-metal or lanthanide complexes, or when breaking covalent bonds. Except in high-spin cases, these systems cannot be accurately described by a single Slater determinant. As a result, widely used single-reference methods like density functional theory (DFT) often fail for such multireference (MR) systems.

To address this, the electronic wave function must be expanded as a linear combination of orthogonal functions, e.g., Slater determinants or configuration state functions (CSFs). This configuration interaction (CI)-based ansatz forms the foundation of all MR methods.

One of the simplest MR approaches is the multiconfigurational self-consistent field (MCSCF) method. MCSCF employs a CI expansion of the wave function and variationally optimizes both molecular orbitals and CI coefficients. A notable advantage of CI wave functions is their direct applicability to excited states: the coefficients for the *n*-th excited state can be obtained from the *n*-th eigenvector of the CI matrix, i.e., the electronic Hamiltonian projected onto the CSF basis.

Excited-state MCSCF methods are affected by additional complications. In the state-specific (SS) approach,^1^ the energy of each state is minimized independently, which can result in nonorthogonal wave functions for states with the same symmetry and, in some cases, root-flipping during optimization.^2^ A common remedy is the state-averaged (SA) MCSCF method,^2^ which minimizes the average energy of several states using a common set of orbitals—though this may reduce accuracy.

An alternative to CI-based excited-state MCSCF is offered by MCSCF propagator theory, pioneered by Jo̷rgensen, Yeager, Dalgaard, and Olsen.^3–5^ Unlike SA-MCSCF, this formalism enables linear state-specific orbital relaxation. Olsen and Jo̷rgensen later rederived these equations using response function theory,^6^ which provided a rigorous framework for computing a wide range of molecular properties.^7^

A direct implementation of MCSCF excitation energies was introduced in ref 8. and remains available in the DALTON package.^9^ Despite the sophistication of that implementation, MCSCF linear-response calculations were historically limited to medium-sized molecules. To enable large-scale applications, recent developments have leveraged modern integral-decomposition techniques^10^ and hybrid parallelization schemes.^11^

The MCSCF linear-response method requires solving a large generalized eigenvalue problem to obtain the lowest excitation energies. This is typically done using iterative Krylov-subspace solvers, such as generalized versions of the Davidson algorithm,^12,13^ which are well suited for such tasks.^10,14^

The convergence efficiency of the Davidson algorithm is closely tied to the quality of the initial eigenvectors and preconditioners, which are usually derived from diagonal approximations of the matrix.^12,13^ Improved convergence can be achieved by constructing better preconditioners, for example using matrix elements from a lower-cost electronic structure method. Following this approach, Zhou and Parker demonstrated substantial speedups in TDDFT calculations using semiempirical methods for the preconditioner.^15^ Similarly, Koch et al. used coupled-cluster with singles and doubles (CCSD) as a preconditioner for CC with iterative triples (CC3) calculations.^16^

In the absence of suitable low-cost methods, an effective alternative is the two-level preconditioning strategy, originally developed for large-scale CI problems.^17,18^ In this scheme, selected important configurations are treated with a full-matrix preconditioner, while the rest use the diagonal approximation. Two-level strategies have also improved convergence in MCSCF,^19,20^ vibrational CC theory,^21^ and even three-level variants have been explored.^22^

In this work, we focus on enhancing the efficiency of solving the MCSCF linear-response eigenvalue problem. Specifically, we develop a custom Davidson algorithm that applies a two-level strategy to both the initial eigenvector guess and the preconditioner. Analogous to two-level strategies used in CI theory,^17,18^ we identify important elements of the response vectors for which full Hessian and metric elements are computed and diagonalized. For all remaining components, a diagonal approximation is employed.^12–14^ After presenting the theory and implementation details, we demonstrate the performance gains across a diverse set of molecules, varying in size, active space, and number of excited states.

## 2 Theoretical Methods

### 2.1 Davidson Algorithm

Our goal is to determine the *n* lowest eigenvalues **ω** and corresponding eigenvectors ***X*** of a large matrix ***A***:

AX=Xω 1

For simplicity, we restrict the discussion to real-symmetric matrices.

We further assume that the number of desired roots *n* is small compared to *N*, the dimension of ***A***. Therefore, iterative algorithms are preferable for solving for the lowest eigenvalues and eigenvectors, as they are more efficient in terms of computational time and memory usage.

A standard algorithm widely used in quantum chemistry for such large eigenvalue problems is the Davidson algorithm^12,13^ (DA). It is favored due to its simplicity and rapid convergence, particularly for diagonally dominant matrices.

The DA proceeds as follows:

1. Start by diagonalizing an approximate matrix ***A*** ^0^:
$$A^{0}X^{0}=X^{0}\omega^{0}$$ 2
  Use the *n* eigenvectors {***x***_*i*_^0^} corresponding to the lowest eigenvalues as initial trial vectors {***b***_*i*_}. If necessary, orthogonalize them (e.g., using the Gram–Schmidt procedure).
2. Compute the matrix-vector products (so-called sigma vectors):
$$\sigma_{i}=Ab_{i}$$ 3
3. Solve the eigenvalue problem ([Disp-formula eq1]) in a reduced space of size *M*, spanned by the current set of trial vectors {***b***_*j*_}:
$$A^{\prime }X^{\prime }=X^{\prime }\omega^{\prime }$$ 4
$$A_{ij}^{\prime }=b_{i}^{T}\sigma_{j},\;1\leq i,\;j\leq M$$ 5
  The reduced-space eigenvalues {ω_α_^′^} converge to the exact eigenvalues {ω_α_} as the space expands. For simplicity, primes will be dropped in the following for all eigenvalues.
4. Construct the full-space eigenvectors {***x***_α_} for each desired root α:
$$x_{\alpha }=\sum_{i}b_{i}x_{i\alpha }^{\prime }$$ 6
5. Compute the residual vector ***r***_α_:
$$r_{\alpha }=\sum_{i}\sigma_{i}x_{i\alpha }^{\prime }-\omega_{\alpha }x_{\alpha }$$ 7
6. Compare the Frobenius norm of each residual vector with a convergence threshold θ. If ∥ ***r***_α_∥ < θ for all roots α, the algorithm terminates.
7. Otherwise, apply a preconditioner ***M*** to the residuals of the unconverged roots:
$$q_{\alpha }=M(\omega_{\alpha })r_{\alpha }$$ 8
  The preconditioner is based on an approximate matrix ***A*** ^0^:
$$M(\omega_{\alpha })=(A^{0}-\omega_{\alpha }I{)}^{-1}$$ 9
8. Orthogonalize the vectors {***q***_α_} using Gram–Schmidt, ensuring they are orthogonal to one another and to the existing trial vectors. The orthonormalized vectors augment the current basis set {***b***_*i*_}.
9. Continue from step 2.

In most applications, ***A*** is extremely large while *n* remains small. Therefore, sigma-vector evaluation dominates the computational cost per iteration.

The total number of iterations—and thus the total runtime—can be reduced significantly by using a good approximation ***A*** ^0^ for both initial eigenvector generation (eq 2) and preconditioning (eq 9). A better ***A*** ^0^ leads to improved trial vectors (closer to the final ***X***) and a better-conditioned preconditioner:

$$N(\omega_{\alpha })=M(\omega_{\alpha })(A-\omega_{\alpha }I)$$ 10

which acts on the approximate solution:

$$q_{\alpha }=N(\omega_{\alpha })x_{\alpha }$$ 11

This auxiliary matrix ***N*** (ω_α_) is used in the construction of approximate Krylov subspaces.^23,24^

Assuming that ***A*** ^0^ cannot be obtained from a more approximate electronic structure method, a common choice for ***A*** ^0^ is to select only a subset of elements of the full matrix ***A***. A common choice for ***A*** ^0^, particularly in quantum chemistry, is a diagonal approximation:

$$A^{0}=D=\mathrm{diag}(A_{11},A_{22},...,A_{NN})$$ 12

as originally proposed by Davidson.^12,13^

This choice is especially effective for diagonally dominant matrices. It is computationally inexpensive, and the inversion in eq 9 becomes trivial:

$$M_{IJ}(\omega_{\alpha })=(D_{I}-\omega_{\alpha }{)}^{-1}\delta_{IJ}$$ 13

Here, capital indices denote matrix elements (*I*, *J*) or vector components (*I*).

Because ***D*** has the same size as a trial vector ***b***_*i*_, it can be efficiently stored in memory or on disk.

If the ***A*** matrix is not diagonally dominant, as it is often revealed by the presence of (nearly) degenerate states, it is necessary to include nondiagonal blocks of ***A*** when setting up ***A*** ^0^,^17,18,22^ as discussed in the textbook chapter of Malmqvist in the context of configuration-interaction (CI) theory.^18^ For a two-level preconditioner,^18^ each component *I* of a vector belongs to one of the two categories: either primary (P) or secondary (Q). Usually, there is relatively small number of P components with a large contribution to the *n* lowest eigenvalues of ***A*** and, accordingly, a large number of Q components with a minor contribution to **ω**_α_.

The approximate ***A*** ^0^ matrix features the following block structure:

$$A^{0}=\left(\begin{array}{ll} A^{\mathrm{PP}} & 0^{\mathrm{PQ}}\\ 0^{\mathrm{QP}} & D^{\mathrm{QQ}} \end{array}\right)$$ 14

The decoupling between P and Q components in eq 14 is beneficial for setting up both initial eigenvectors {***x***_*i*_^0^} and a preconditioner ***M*** matrix. Due to the zero-coupling blocks, each of the two diagonal blocks PP and QQ can be diagonalized ([Disp-formula eq2]) and shifted-and-inverted ([Disp-formula eq9]) individually:

$$M=\left(\begin{array}{ll} M^{\mathrm{PP}} & 0^{\mathrm{PQ}}\\ 0^{\mathrm{QP}} & M^{\mathrm{QQ}} \end{array}\right)$$ 15

Initial eigenvectors ***x***_α_^0^ for the secondary components Q are available from the unit vectors associated with the *n* smallest elements in ***D*** ^QQ^. For all primary component-matrix elements ***A*** ^PP^, the full eigenvalue equations are solved:

$$A^{\mathrm{PP}}X^{P}=X^{P}\omega^{P}$$ 16

Then, the eigenvectors ***x***_α_^P^ associated with the *n* lowest eigenvalues ω_α_^P^ can be taken as initial eigenvectors for the DA. Depending on the number of P components, Q components, and the number of desired roots *n*, the set of initial eigenvectors may comprise of both P-space and Q-space vectors.

Our general strategy to divide individual vector elements into the P and Q space is the following:

- First, the diagonal approximation $D_{A}$ to the full ***A*** matrix is made for all vector elements.
- Then, we search for the *N*_P_ smallest elements in $D_{A}$ and store their index in the full space.
- Finally, all remaining vector elements are associated with the secondary (Q) space. Their full-space index is memorized as well.

Note that the number of P-space elements *N*_P_ is an input parameter and is limited by the cost for evaluating the ***A*** matrix in P space, ***A*** ^PP^, and solving the P-space eigenvalue eq 16. Typical P-space dimensions are in the range of 200–2000, though depend strongly on the type of eigenvalue equations to be solved.

The preconditioner matrix ***M*** ^QQ^ of the QQ block is readily available by applying eq 13 to all secondary components. For the PP block, the shifted inverse

$$M^{\mathrm{PP}}(\omega_{\alpha })=X^{P}(\omega^{P}-\omega_{\alpha }I^{\mathrm{PP}}{)}^{-1}(X^{P}{)}^{T}$$ 17

can be computed conveniently in the spectral representation from the diagonalized ***A*** ^PP^ block ([Disp-formula eq16]) that has been generated previously for the initial eigenvectors.

In the case where all components fall in P space, the initial guesses already diagonalize ***A***, and the residuals vanish. New trial vectors {***q***_α_} would then be linearly dependent and contribute no new direction.

To avoid this, Olsen proposed a quasi-Newton (QN) correction:^17^

$$q_{\alpha }^{\mathrm{QN}}=-M(\omega_{\alpha })\left(r_{\alpha }-\frac{x_{\alpha }^{T}M(\omega_{\alpha })r_{\alpha }}{x_{\alpha }^{T}M(\omega_{\alpha })x_{\alpha }}x_{\alpha }\right)$$ 18

which ensures orthogonality of the new trial vector to the current solution.

### 2.2 Generalized Linear Response Eigenvalue Problem

For variational electronic structure methods—such as self-consistent field (SCF) approaches, including Hartree–Fock (HF), Kohn–Sham density functional theory (DFT), and multiconfigurational SCF (MCSCF), the perturbed wave function (WF) is expressed in terms of excitation and deexcitation operators.

This dual nature leads to a structured eigenvalue problem of the form:

$$\left(\begin{array}{cc} A & B\\ B^{*} & A^{*} \end{array}\right)\left(\begin{array}{c} X\\ Y^{*} \end{array}\right)=\left(\begin{array}{cc} \Sigma & \Delta \\ -\Delta^{*} & -\Sigma^{*} \end{array}\right)\left(\begin{array}{c} X\\ Y^{*} \end{array}\right)\omega$$ 19

which is solved for the excitation energies **ω**.

For HF wave function, this formulation is commonly referred to as the random phase approximation (RPA).^25–27^

In the remainder of this article, we restrict ourselves to the nonrelativistic case and real-valued wave functions, treating all quantities in eq 19 as real.

Neglecting the coupling blocks ***B*** and **Δ** leads to a simplified form:

AX=ΣXω 20

known as the Tamm–Dancoff approximation (TDA), which is commonly used in time-dependent DFT (TDDFT) calculations.^28^

A key feature of the RPA eigenvalue problem ([Disp-formula eq19]) is the pairing of its 2*N* eigenvalues as ±**ω**. Thus, only the *N* positive excitation energies and corresponding eigenvectors need to be computed. The negative roots (deexcitation energies) follow directly by interchanging the eigenvector components:

$$\left[\left(\begin{array}{cc} A & B\\ B^{*} & A^{*} \end{array}\right)+\omega \left(\begin{array}{cc} \Sigma & \Delta \\ -\Delta^{*} & -\Sigma^{*} \end{array}\right)\right]\left(\begin{array}{c} Y\\ X \end{array}\right)=\left(\begin{array}{c} 0\\ 0 \end{array}\right)$$ 21

When applying the Davidson algorithm to eq 19, each root must be represented by two trial vectors in order to maintain the dual structure of the eigenvalue problem in the reduced space.^14^

As in all generalized eigenvalue problems, the eigenvectors in eq 19 require a suitable normalization. Following Olsen et al.,^14^ we normalize the eigenvectors to one over the metric:

$${\left(\begin{array}{c} X\\ Y \end{array}\right)}^{T}\left(\begin{array}{cc} \Sigma & \Delta \\ -\Delta & -\Sigma \end{array}\right)\left(\begin{array}{c} X\\ Y \end{array}\right)=I$$ 22

Note that the norm becomes −***I*** for the interchanged eigenvectors in eq 21.

We favor a reformulation of eq 19 based on the linear combinations of the upper and lower part:

$$\left(\begin{array}{cc} A+B & 0\\ 0 & A-B \end{array}\right)\left(\begin{array}{c} X^{+}\\ X^{-} \end{array}\right)=\left(\begin{array}{cc} 0 & \Sigma -\Delta \\ \Sigma +\Delta & 0 \end{array}\right)\left(\begin{array}{c} X^{+}\\ X^{-} \end{array}\right)\omega$$ 23

This representation is more consistent with the formalism of perturbation theory for time-independent molecular properties,^29^ in which the ***A*** ∓ ***B*** matrices appear in response equations for electric and magnetic properties.^30^

To retain the normalization in eq 22, the transformation:

$$X^{\pm }=\frac{1}{\sqrt{2}}(X\pm Y)$$ 24

must be applied.

The deexcitation energies −**ω** can be retrieved by flipping the sign of the lower component:

$$\left[\left(\begin{array}{cc} A+B & 0\\ 0 & A-B \end{array}\right)+\omega \left(\begin{array}{cc} 0 & \Sigma -\Delta \\ \Sigma +\Delta & 0 \end{array}\right)\right]\left(\begin{array}{c} X^{+}\\ -X^{-} \end{array}\right)=\left(\begin{array}{c} 0\\ 0 \end{array}\right)$$ 25

as can be verified using eq 24.

To compute the lowest roots of the reformulated RPA problem in eq 23 using the Davidson algorithm, two sets of trial vectors are required to represent the components ***X*** ^±^ of each root.^14^

Further algorithmic details, including residual vector computation and orthonormalization, can be found in our previous work.^10^

### 2.3 A Block Preconditioner for Linear-Response Eigenvalue Equations

To reduce the number of iterations needed to solve the TDA eq 20, initial eigenvectors are generated by solving an approximate version of eq 20:

$$A^{0}X^{0}=\Sigma^{0}X^{0}\omega^{0}$$ 26

Here, ***A*** ^0^ and **Σ** ^0^ are approximations to the full Hessian and metric matrices, respectively.

The corresponding preconditioner is the inverse of the matrix pencil (***A*** ^0^, **Σ** ^0^):

$$M(\omega_{\alpha })=(A^{0}-\omega_{\alpha }\Sigma^{0}{)}^{-1}$$ 27

This inverse can be conveniently expressed in its spectral representation using the eigenvalues and eigenvectors from eq 26:

$$M(\omega_{\alpha })=X^{0}(\omega^{0}-\omega_{\alpha }I{)}^{-1}(X^{0}{)}^{T}$$ 28

assuming the eigenvectors are normalized over the metric to one:

$$(X^{0}{)}^{T}\Sigma^{0}X^{0}=I$$ 29

The validity of eq 28 can be confirmed by evaluating:

$$\begin{array}{cc} N^{0}(\omega_{\alpha }) & =M(\omega_{\alpha })(A^{0}-\omega_{\alpha }\Sigma^{0})\\ & =X^{0}(\omega^{0}-\omega_{\alpha }I{)}^{-1}(X^{0}{)}^{T}(A^{0}-\omega_{\alpha }\Sigma^{0})\\ & =X^{0}(X^{0}{)}^{-1}=I \end{array}$$ 30

In our two-level block preconditioner, we use the spectral form (eq 28) of the inverse pencil (***A*** ^P^, **Σ** ^P^) for the primary space:

$$M(\omega_{\alpha }{)}^{\mathrm{PP}}=X^{P}(\omega^{P}-\omega_{\alpha }I^{\mathrm{PP}}{)}^{-1}(X^{P}{)}^{T}$$ 31

For the secondary space, we approximate ***A*** and **Σ** with their diagonal elements, leading to

$$M_{IJ}^{\mathrm{QQ}}(\omega_{\alpha })=(A_{II}-\omega_{\alpha }\Sigma_{II}{)}^{-1}\delta_{IJ}$$ 32

For RPA-type equations, a similar reduction in DA iterations is possible.

Initial eigenvectors are obtained by solving the approximate version of the reformulated RPA eq 23 using approximate blocks ***A*** ^0^, ***B*** ^0^, and **Σ** ^0^, **Δ** ^0^:

$$\left(\begin{array}{cc} A^{0}+B^{0} & 0\\ 0 & A^{0}-B^{0} \end{array}\right)\left(\begin{array}{c} X^{0,+}\\ X^{0,-} \end{array}\right)=\left(\begin{array}{cc} 0 & \Sigma^{0}-\Delta^{0}\\ \Sigma^{0}+\Delta^{0} & 0 \end{array}\right)\left(\begin{array}{c} X^{0,+}\\ X^{0,-} \end{array}\right)\omega^{0}$$ 33

Consistent with the two-level block strategy in eq 14, this system is solved only for elements belonging to the primary space. The resulting eigenvectors {***x***_α_^P,+^} and {***x***_α_^P,–^} associated with the lowest *n* eigenvalues {ω_α_^P^} serve as initial trial vectors for the Davidson algorithm.^10^

The inverse of the RPA matrix pencil, used for preconditioning in P space, can be written as

$$M^{\mathrm{PP}}=\left(\begin{array}{cc} X^{P,+} & X^{P,+}\\ X^{P,-} & -X^{P,-} \end{array}\right)\left(\begin{array}{cc} (\omega^{P}-\omega_{\alpha }I^{\mathrm{PP}}{)}^{-1} & 0\\ 0 & (\omega^{P}+\omega_{\alpha }I^{\mathrm{PP}}{)}^{-1} \end{array}\right){\left(\begin{array}{cc} X^{P,+} & X^{P,+}\\ X^{P,-} & -X^{P,-} \end{array}\right)}^{T}$$ 34

Note that the denominator (**ω** ^P^ + ω_α_) differs in sign from standard Green’s function expressions,^29^ due to the −***I*** normalization of the negative-energy eigenvectors.

For the secondary space, we again employ a diagonal approximation to construct the preconditioner:

$$M^{\mathrm{QQ}}=\frac{1}{2}\left(\begin{array}{cc} I^{\mathrm{QQ}} & I^{\mathrm{QQ}}\\ I^{\mathrm{QQ}} & -I^{\mathrm{QQ}} \end{array}\right)\left(\begin{array}{cc} (D_{A}-\omega_{\alpha }D_{\Sigma }{)}^{-1} & 0\\ 0 & (D_{A}+\omega_{\alpha }D_{\Sigma }{)}^{-1} \end{array}\right)\left(\begin{array}{cc} I^{\mathrm{QQ}} & I^{\mathrm{QQ}}\\ I^{\mathrm{QQ}} & -I^{\mathrm{QQ}} \end{array}\right)$$ 35

Applying this Q-space preconditioner to the residual vectors {***r***_α_^+^} and {***r***_α_^–^} yields

$$M^{\mathrm{QQ}}\left(\begin{array}{c} r^{+}\\ r^{-} \end{array}\right)=((D_{A}-\omega_{\alpha }D_{\Sigma })(D_{A}+\omega_{\alpha }D_{\Sigma }){)}^{-1}\left(\begin{array}{c} D_{A}r^{+}+\omega_{\alpha }D_{\Sigma }r^{-}\\ \omega_{\alpha }D_{\Sigma }r^{+}+D_{A}r^{-} \end{array}\right)$$ 36

which is in full agreement with previous works:^10,31^

### 2.4 CASSCF Linear Response Theory

As in configuration interaction (CI) theory, the MCSCF wave function for a single state |0⟩ is expressed as a linear combination of configuration state functions (CSFs) |Φ_*I*_⟩:

$$\left|0\rangle =\sum_{I}C_{I}|\Phi_{I}\right\rangle$$ 37

The complete active space SCF (CASSCF) model is a special case of MCSCF, in which the CSFs are generated by distributing a fixed number of electrons among a selected set of valence (active) orbitals in all possible ways.^32^

A schematic of the CASSCF molecular orbital (MO) partitioning—including core (inactive), active, and virtual orbitals—is shown in Figure 1 with associated index labels.

**Figure 1:**
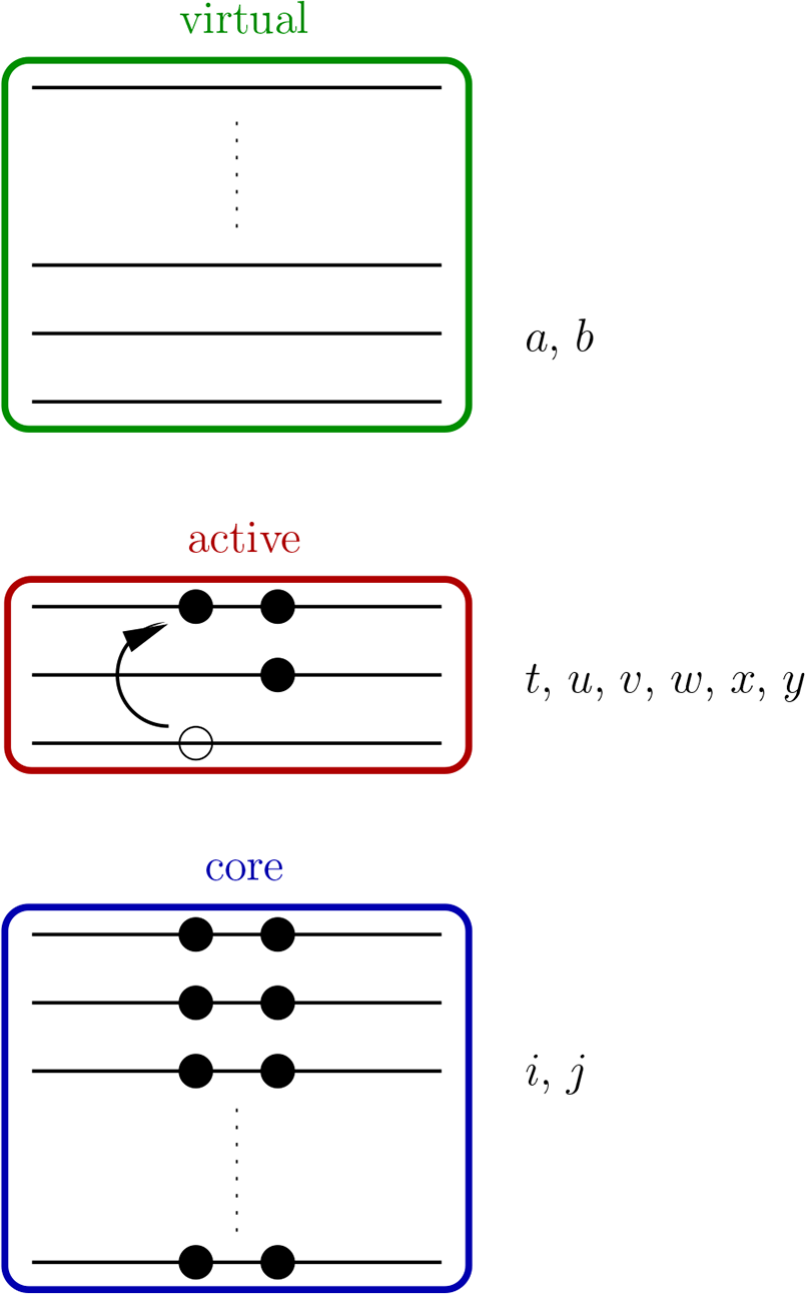
Schematic CASSCF MO diagram showing default index labels for core (inactive), active, and virtual MO subspaces.

The CASSCF energy is determined by minimizing the expectation value of the electronic Hamiltonian:

E=minκ,s⟨0|H^|0⟩ 38

with respect to orbital **κ** and configuration variational parameters ***S***.

This fully variational formulation is well suited for computing molecular properties.^7,33^

The Hamiltonian in eq 38 is the nonrelativistic Born–Oppenheimer Hamiltonian, expressed in second quantization as

H^=∑pqhpqE^pq+12∑pqrs(pq|rs)e^pqrs 39

e^pqrs=E^pqE^rs−δqrE^ps 40

where *Ê*_*pq*_ denotes spin-summed excitation operators:

E^pq=a^pα†a^qα+a^pβ†a^qβ 41

Throughout this work, *p*, *q*,... represent general MO indices.

In variational wave function models, the time-dependent wave function |0̃⟩ (in phase-isolated form) can be written as a unitary transformation of the ground-state reference:^7,33^

|0~⟩=exp(Λ^(t))|0⟩ 42

The operator Λ̂(*t*) is anti-Hermitian and decomposes into orbital and configuration parts:

Λ^(t)=Λ^o(t)Λ^c(t) 43

Λ^o(t)=∑m>0(κm(t)q^m†−κm*(t)q^m) 44

Λ^c(t)=∑m>0(Sm(t)R^m†−Sm*(t)R^m) 45

Here, *q̂*_*m*_^†^ and *q̂*_*m*_ denote orbital excitation and deexcitation operators:

q^m†=E^pqandq^m=E^qp 46

state-transfer operators *R̂*_*m*_^†^, exciting from the CASSCF reference state |0⟩ to excited state of the CAS-CI Hamiltonian |*m*⟩, and the complex conjugate partner *R̂*_*m*_ are used for the configuration part:

R^m†=|m⟩⟨0|andR^m=|0⟩⟨m| 47

In practice, it is more efficient to use an orthogonal complement space {|*m*⟩} = {|Φ_*I*_⟩}\|0⟩ spanned by CSFs and the reference state projected out.^6^ This avoids explicit diagonalization of the full CAS-CI Hamiltonian.

The dual nature of excitations and deexcitations in the CASSCF model leads naturally to a generalized RPA-type eigenvalue problem, as introduced in Section 2.2. Moreover, due to the presence of two variational parameters—orbital and CI coefficients—the multiconfigurational RPA (MCRPA) Hessian and metric matrices acquire an additional two-by-two block structure:

Amn=(⟨0|[q^m,[H^,q^n†]]|0⟩⟨0|[[q^m,H^],R^n†]|0⟩⟨0|[R^m,[H^,q^n†]]|0⟩⟨0|[R^m,[H^,R^n†]]|0⟩)Bmn=(⟨0|[q^m,[H^,q^n]]|0⟩⟨0|[[q^m,H^],R^n]|0⟩⟨0|[R^m,[H^,q^n]]|0⟩⟨0|[R^m,[H^,R^n]]|0⟩)Σmn=(⟨0|[q^m,q^n†]|0⟩⟨0|[q^m,R^n†]|0⟩⟨0|[R^m,q^n†]|0⟩⟨0|[R^m,R^n†]|0⟩)Δmn=(⟨0|[q^m,q^n]|0⟩⟨0|[q^m,R^n]|0⟩⟨0|[R^m,q^n]|0⟩⟨0|[R^m,R^n]|0⟩) 48

These matrices are necessary in the general MCSCF linear response framework, which includes orbital rotations among active orbitals. However, for the CASSCF model, such active–active rotations are redundant, as they are already captured by the CI wave function.

As a result, several simplifications apply for CASSCF:

- Active–active blocks in the orbital rotation space are zero.
- The entire **Δ** matrix vanishes.
- The off-diagonal blocks of the metric matrix **Σ**—i.e., ⟨0|[*q̂*_*m*_,*R̂*_*n*_^†^]|0⟩ and ⟨0|[*R̂*_*m*_,*q̂*_*n*_^†^]|0⟩—are zero.

Efficient implementation of the sigma-vector computation for MCRPA has been discussed previously.^8,10,11^ In the remainder of this section, we focus on constructing the Hessian matrix elements ***A*** and ***B*** explicitly in the primary space P (i.e., a selected subset of orbital and configuration vectors).

Explicit expressions for the ***A***– ***B*** supermatrix elements have previously been reported for the orbital–orbital blocks.^34,35^ Additionally, we provide here the corresponding equations for the ***A*** + ***B*** supermatrix, summarized in Table 1. These expressions facilitate their use in the preconditioning of linear response equations involving purely imaginary magnetic perturbations,^36–38^ and, more importantly in the present context, in the construction of both the initial guess and the preconditioner for the MCRPA equations. Explicit expressions for the TDA supermatrix ***A*** is readily available by omitting all terms succeeding the ± signs.

**Table 1:** Explicit Equations for the CASSCF Orbital–Orbital Hessian ***A*** ± ***B*** and Metric Matrix **Σ**

$$\begin{array}{cc} (A\pm B{)}_{ai,bj} & =2((f_{ab}^{I}+f_{ab}^{A})\delta_{ij}-(f_{ji}^{I}+f_{ji}^{A})\delta_{ab}\\ & +(2\mp 2)(ai|jb)-(ab|ji)\pm (aj|bi)) \end{array}$$
$$\begin{array}{cc} (A\pm B{)}_{ti,bj} & =\delta_{ij}\left(2f_{bt}^{I}-\sum_{x}f_{bx}^{I}D_{xt}-Q_{bt}+2f_{tb}^{A}\right)\\ & +\sum_{x}(2\delta_{tx}-D_{xt})((2\mp 2)(xi|jb)-(ji|xb)\pm (xj|bi)) \end{array}$$
$$\begin{array}{cc} (A\pm B{)}_{ti,uj} & =\delta_{ij}\left(2f_{ut}^{I}-\sum_{x}f_{ux}^{I}D_{xt}+2f_{tu}^{A}-Q_{ut}\right)\\ & -\delta_{tu}(2f_{ji}^{I}+2f_{ji}^{A})+f_{ji}^{I}D_{ut}\\ & +\sum_{x}(\delta_{tx}-D_{xt})((2\mp 2)(xi|ju)-(ji|xu)\pm (xj|ui))\\ & +\sum_{x}(\delta_{ux}-D_{ux})(2(ti|jx)-(ji|tx))\\ & \pm \sum_{x}(2(ti|xj)-(xi|tj))D_{xu}\\ & +\sum_{xy}((jy|xi)(d_{xtuy}\mp d_{xtyu})+(ji|xy)d_{utxy}) \end{array}$$
$$\begin{array}{cc} (A\pm B{)}_{at,bj} & =-\delta_{ab}\left(\sum_{x}f_{jx}^{I}D_{tx}+Q_{it}\right)\\ & +\sum_{x}((2\mp 2)(ax|jb)-(ab|jx)\pm (bx|aj))D_{tx} \end{array}$$
$$\begin{array}{cc} (A\pm B{)}_{at,uj} & =\sum_{x}((2\mp 2)(ax|ju)-(au|jx)\pm (aj|ux))D_{tx}\\ & -\sum_{xy}(ax|jy)(d_{txuy}\mp d_{txyu})\\ & \pm \left(f_{aj}^{I}D_{tu}+\sum_{xy}(aj|xy)d_{tuxy}\right) \end{array}$$
$$\begin{array}{cc} (A\pm B{)}_{at,bu} & =\delta_{ab}\left(-Q_{ut}-\sum_{x}f_{ux}^{I}D_{tx}\right)+f_{ab}^{I}D_{tu}\\ & +\sum_{xy}((ab|xy)d_{tuxy}+(ax|yb)(d_{txyu}\mp d_{txuy})) \end{array}$$
$$\Sigma_{ai,bj}=2\delta_{ab}\delta_{ij}$$
$$\Sigma_{ti,uj}=(2\delta_{tu}-D_{ut})\delta_{ij}$$
$$\Sigma_{at,bu}=\delta_{ab}D_{tu}$$

The matrix elements in Table 1 depend on the one- and two-body reduced density matrices:

Dtu=⟨0|E^tu|0⟩ 49

dtuvw=⟨0|e^tuvw|0⟩ 50

Additional intermediates include inactive (I) and active (A) Fock matrices:

$$f_{pq}^{I}=h_{pq}+\sum_{j}(2(pq|jj)-(pj|jq))$$ 51

$$f_{pq}^{A}=\sum_{vw}\left(\left(pq|vw)-\frac{1}{2}(pw|vq\right)\right)D_{vw}$$ 52

and transformed two-electron integral terms:

$$Q_{pu}=\sum_{wxy}(pw|xy)d_{uwxy}$$ 53

These intermediates are also used in standard CASSCF electronic gradient calculations^32^ and are computationally efficient to obtain.^39^

Applying the diagonal approximation ***D***_A_ to ***A*** directly retains two-electron integrals, e.g., for the occupied-virtual orbital blocks

$$D_{A_{ai}}^{\prime }=2\left(f_{aa}^{I}+f_{aa}^{A}\right)-2\left(f_{ii}^{I}+f_{ii}^{A}\right)+4\left(ai|ia\right)-2(aa|ii)$$ 54

which can be costly when evaluated for all orbital rotations.

To avoid this cost, we follow Chaban et al.^40^ and approximate these terms using only active Fock elements and 1-RDM as shown in Table 2.

**Table 2:** Approximate Diagonal Elements of the Orbital–Orbital Hessian ***D***_**A** ^o^_

$$D_{A_{ai}}=2(f_{aa}^{I}+f_{aa}^{A})-2(f_{ii}^{I}+f_{ii}^{A})$$
$$\begin{array}{cc} D_{A_{ti}} & =2(f_{tt}^{I}+f_{tt}^{A})-\sum_{x}f_{tx}^{I}D_{xt}-Q_{tt}\\ & -2(f_{ii}^{I}+f_{ii}^{A})+(f_{ii}^{I}+f_{ii}^{A})D_{tt} \end{array}$$
$$D_{A_{at}}=-\sum_{x}f_{tx}^{I}D_{tx}-Q_{tt}+(f_{aa}^{I}+f_{aa}^{A})D_{tt}$$

These diagonal approximations are computationally inexpensive and typically positive-definite, making them suitable for preconditioning.

Only the ***A*** matrix contributes to the configuration-configuration Hessian. ***A*** ^PP^ is computed using a subset of the CSF basis:

AIJ=⟨ΦI|H^|ΦJ⟩−EδIJ 55

with the CI matrix evaluated as

⟨ΦI|H^|ΦJ⟩=EcδIJ+∑tuhtu′AIJtu+12∑tuvw(tu|vw)∑KAIKtuAKJvw 56

where:

$$E^{c}=V_{N}+\sum_{i}(h_{ii}+f_{ii}^{I})$$ 57

$$h_{tu}^{\prime }=f_{tu}^{I}-\frac{1}{2}\sum_{v}(tv|vu)$$ 58

The coupling coefficients^41^ *A*_*IJ*_^*tu*^ = ⟨Φ_*I*_| *Ê*_*tu*_|Φ_*J*_⟩ are computed using standard algorithms based on bonded functions.^42,43^

The diagonal elements for the configuration part are

DAI=⟨ΦI|H^|ΦI⟩−E 59

Our implementation uses a nonredundant orthogonal complement basis for the CI part of the response vectors, with an efficient transformation between the redundant and nonredundant CSF spaces.^6,44^

To decouple orbital and configuration blocks for preconditioning, we neglect the off-diagonal Hessian blocks. This enables independent treatment of each space and is essential in SA-CASSCF response theory,^35,44,45^ where the number of CI matrix elements grows with the number of averaged states. Such decoupling also permits the use of larger P-space dimensions within each subspace, thereby improving preconditioner quality and accelerating convergence.

The primary-space orbital pairs are chosen from the *N*_P_ smallest elements of ***D***_A^o^_ (Table 2). The configuration part uses the *N*_P_ lowest diagonal matrix elements *D*_A_*I*__ from the diagonal CI matrix.

To reduce integral transformation cost for the P-space Hessian matrix elements, we apply the resolution-of-the-identity (RI) approximation^46–49^ using Coulomb-fitting^50^

$$V_{\mathrm{PQ}}=\left(\chi_{P}|\frac{1}{r_{12}}|\chi_{Q}\right)$$ 60

with auxiliary atomic basis functions χ_P_ and χ_Q_.

For core–virtual orbital pairs in the P space, the integrals are computed via:

$$(P|\mu i)=\sum_{\nu }(P|\mu \nu )C_{\nu i}$$ 61

$$(P|ai)=\sum_{\mu }(P|\mu i)C_{\mu a}$$ 62

$$B_{ai}^{Q}=\sum_{P}[V^{-1/2}{]}_{\mathrm{PQ}}(P|ai)$$ 63

$$(ai|bj)=\sum_{Q}B_{ai}^{Q}B_{bj}^{Q}$$ 64

Integrals of other orbital pairs are transformed accordingly.

If we assume that number of MOs needed for the orbital pairs in P space is constant in practical calculations, and if the Cauchy–Schwarz screening of orbital basis-function pairs is employed, both the full four-center and the RI integral transformation scale quadratically with the system size. Though, the prefactor of the RI integral transformation is usually at least 1 order of magnitude faster depending on the system size.^51^

When RI is used also for the sigma-vector integral transformation,^10,44^ the same auxiliary basis is used for constructing P-space integrals (Table 1). In that case, the JK auxiliary basis^52^ is most used for integrals occurring in the sigma-vector and P-space Hessian computation. If the sigma vectors are computed without the RI approximation, we use the smaller C auxiliary basis sets^51^ for the P-space Hessian.

## 3 Results and Discussion

All calculations were performed using ORCA^53^ version 6.1. CASSCF linear-response calculations employing diagonal Hessians for both the initial guess and preconditioning (denoted as Diag/Diag) were carried out with a development version of ORCA (Git hash 1e25dd27). This feature will be available in the next official release. In contrast, ORCA 6.0 used two-level approximations for the Hessian and metric in the initial guess, while still employing diagonal preconditioning (denoted as Full/Diag). All calculations were executed in parallel mode using 16 processes spawned via the Message Passing Interface (MPI).

All CASSCF ground-state calculations were initialized using molecular orbitals (MOs) obtained via the AVAS procedure,^54^ based on a prior BP86 DFT calculation.^55,56^ The energy equations were converged using the step-restricted second-order converger TRAH.^44^

All MCTDA and MCRPA calculations used a convergence threshold of 10^–5^, evaluated via the Frobenius norm of the residual vector for each root. The reduced subspace in the Davidson algorithm (DA) was limited to 200 vectors. If this limit was reached, the DA was restarted using the current eigenvectors. The P-space for each of the two response-vector blocks (orbital and configuration) was limited to a size of 400 in all CASSCF linear-response calculations.

We selected four molecular systems from previous studies, shown in Figure 2, covering a range of molecular sizes, active-space dimensions, and numbers of excited states.

**Figure 2:**
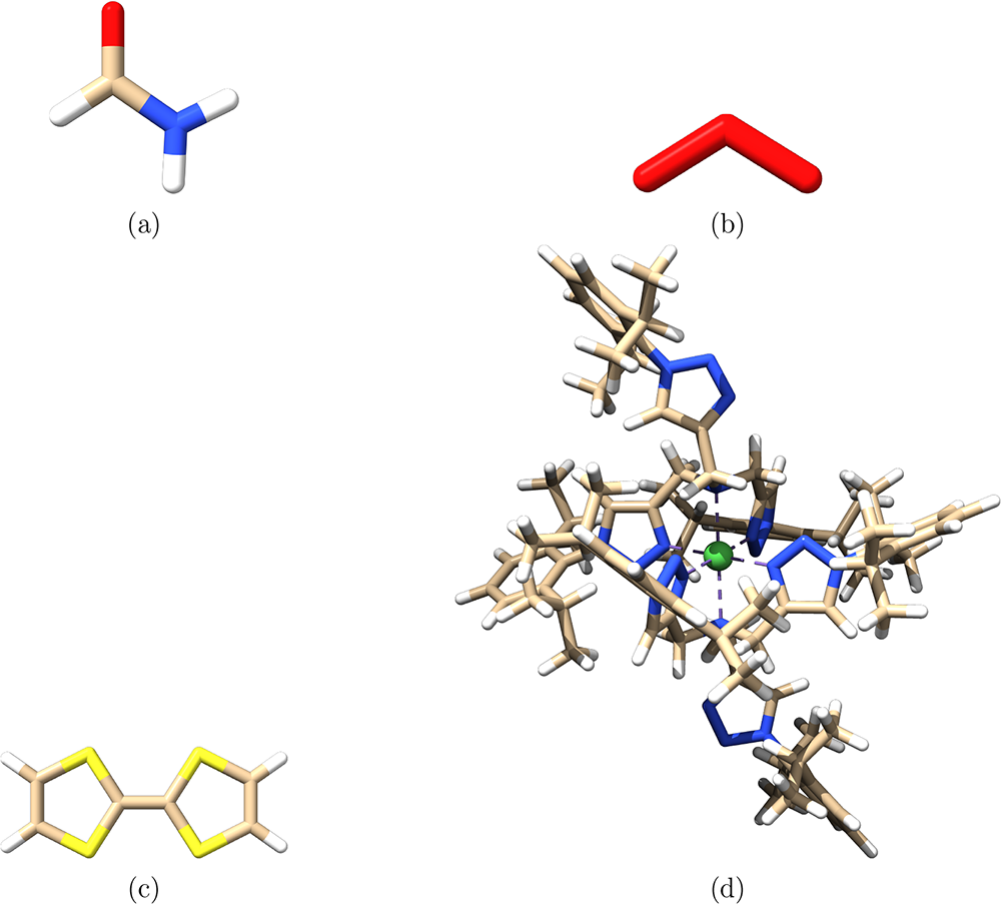
Molecular structures: (a) formamide, (b) ozone (O_3_), (c) tetrathiafulvalene monocation (TTF^+^), and (d) Ni^II^ (pyta)_3_^2+^ complex.

The structure of formamide (A) was taken from ref 57. Calculations employed the TZVP basis set,^58^ and RI approximation was used for the P-space Hessian elements, with AutoAux-generated auxiliary basis sets.^59^ Core 1s orbitals of C, N, and O were frozen for the CASSCF-LR calculation. The active space included π and π* orbitals: CAS­(4,3). The ground state is the totally symmetric singlet state 1 A’.

For ozone (B), we used a *C*_2v_-symmetric structure from ref 60. with bond length 1.278 Å and bond angle 116.8°. Calculations used the aug-cc-pVQZ-DK basis set^61–63^ and scalar-relativistic Douglas–Kroll–Hess DKH Hamiltonian.^64,65^ RI and AutoAux were used as above. Valence (B1) and core (B2) excitations were computed using a CAS­(12,9). For B1, O 1s orbitals were frozen; B2 used the CVS approximation^60^ without configuration response in an all-electron calculation. All LR calculations started from the ^1^A_1_ ground state.

The structure of tetrathiafulvalene monocation (TTF^+^ , C) was taken from ref 10. We used the def2-TZVP orbital basis^66^ and def2/JK auxiliary sets.^52,67^ RIJ was used for Coulomb builds,^68^ and RI was also used in integral transformations for σ vectors and P-space Hessians.^10,39,44^ Exchange was treated seminumerically via COSX^69–71^ with grid def-Grid3.^71^ We froze C 1s and S 1s2s2p orbitals. The active space included all π/π* orbitals: CAS­(13,10). LR calculations started from the 2 B_3u_ ground state.

Calculations for molecules A–C were performed on a single Intel Haswell node (Intel Xeon CPU E5-2687W v3 3.10 GHz), using 4.0 GB of memory for systems A and B, and 6.0 GB for system C.

The final molecule (D) is the Ni II (pyta)_3_^2+^ complex, with structure from ref 72. Calculations used the def2-SVP basis set,^66^ with RIJCOSX and RI-transformation analogous to system C. Core orbitals (1s of C, N; 1s2s2p of Ni) were frozen. Two active-space variants were used: (D1) ROHF with d_*z* ^2^_ and d_*x* ^2^–*y* ^2^_ active orbitals in a triplet state; (D2) CAS­(10,11) double-shell including a σ-bonding d_*x* ^2^–*y* ^2^_ orbital. Calculations used 9.6 GB per MPI process on a single node with an AMD EPYC 7302 processor.

Details on the number of roots and the dimensions of the orbital and configuration response vectors for each system are summarized in Table 3.

**Table 3:** Number of Roots and Dimension of the Orbital and Configuration Response Vectors^a^

calc.	molecule	roots	dim. orb.	dim. cfg.
A	formamide	5	641	5
B1	O_3_ (val.)	25	2727	2519
B2	O_3_ (core)	15	702	0
C	TTF^+^	50	8977	13,859
D1	Ni(pyta)_3_^2+^ (ROHF)	18	525,937	0
D2	Ni(pyta)_3_^2+^ (σ,3d,4d)	18	535,548	98,009

a For further details see text.

The aim of this work is to reduce the number of expensive sigma-vector computations in CASSCF linear-response calculations by providing improved initial eigenvectors and preconditioners. To that end, we introduced a two-level block strategy for the Hessian elements used in the initial guess and preconditioning, as described in Section 2.

To evaluate the performance of this strategy across the six systems introduced before, we employed the following combinations of initial eigenvectors and preconditioners:

**Diag/Diag**: both initial eigenvectors and preconditioner from the diagonal Hessian.
**Full/Diag**: two-level block strategy for initial eigenvectors; diagonal approximation for preconditioning.
**Full/Full**: two-level block strategy for both initial eigenvectors and preconditioner.
**Full/Olsen**: two-level block strategy for both, combined with Olsen’s method for trial vectors.

The number of sigma vectors required to converge MCTDA and MCRPA calculations is shown in Table 4, and the total computation times in Table 5.

**Table 4:** Number of Sigma Vectors to Converge the MCTDA and MCRPA Eigenvalue Calculations^a^

	diag/diag	full/diag	full/full	full/Olsen
MCTDA				
A	54	36	17	20
B1	615	467	322	328
B2	118	81	58	58
C	1939	1122	865	806
D1	379	315	264	263
D2	534	365	307	307
MCRPA				
A	54	51	21	22
B1	649	509	395	403
B2	120	84	60	60
C	2043	1487	1088	1070
D1	438	341	320	322
D2	636	499	410	413

a Different combinations of initial Hessians/preconditioners are given: diag—diagonal Hessian; full—two-level Hessian blocks. For further details see text.

**Table 5:** Total Timings (min) for Solving the MCTDA and MCRPA Eigenvalue Calculations^a^

	diag/diag	full/diag	full/full	full/Olsen	speedup
MCTDA					
A	0.086	0.073	0.046	0.050	1.87
B1	35.98	27.05	19.25	20.47	1.87
B2	7.059	5.103	4.030	4.004	1.75
C	82.14	51.59	40.09	38.85	2.05
D1	1026	902.2	742.7	730.6	1.38
D2	1496	1123	915.7	906.5	1.65
MCRPA					
A	0.186	0.195	0.129	0.135	1.44
B1	51.20	39.92	32.52	33.60	1.57
B2	9.722	7.190	5.683	5.621	1.71
C	95.93	70.32	54.27	54.93	1.77
D1	1194	959.9	914.0	914.0	1.31
D2	1877	1507	1279	1283	1.47

a For the speedup calculations, we compare diagonal Hessian (diag/diag) and the two-level block Hessian approach for both the initial eigenvector and the preconditioner (full/full). Further details are given in the text.

Unsurprisingly, the most approximate scheme (Diag/Diag) results in the highest iteration count across most systems. These runs are typically the slowest to converge, with the exception of the MCRPA calculation on the smallest system (A). For that case, Full/Diag required fewer sigma vectors but slightly more time (5%) due to overhead from constructing and diagonalizing the P-space Hessian — exacerbated by a complex root encountered during iteration, which disappeared at final convergence.

Using the two-level strategy only for initial eigenvectors (Full/Diag) reduced both sigma-vector counts and overall computation times except for the MCRPA calculation on the smallest system (A). Speedups ranged from 1.14 for the large ROHF-TDA calculation (D1) to 1.59 for the medium-sized MCTDA system (C).

Applying the two-level strategy throughout (Full/Full) yielded systematic reductions in runtime, with speedups of 1.05 (MCRPA, D1) to 1.59 (MCTDA, A) relative to Full/Diag. The performance gain is expected, as the cost of constructing and diagonalizing the P-space Hessian is already paid in Full/Diag, and its reuse in preconditioning is effectively free. Thus, we strongly recommend the Full/Full setup when approximate beyond-diagonal Hessians are available.

Using Olsen’s method on top of Full/Full did not lead to consistent improvements. Half of the systems showed negligible gains (max speedup of 1.03 for MCTDA with system C), while the rest experienced slight slowdowns due to increased sigma-vector counts or costlier preconditioning steps (cf. eq 18). As a result, the default in ORCA 6.1 remains Full/Full. In the future, we may trigger the Olsen update automatically for many-root calculations, though with the current data identifying reasonable trigger points is not evident.

Table 5 summarizes total speedups for Full/Full relative to Diag/Diag. As expected, RPA-type equations converged more slowly than their TDA counterparts and generally required more time. Nevertheless, due to the efficient RPA sigma-vector implementation,^10^ the MCRPA runs were typically less than twice as slow as MCTDA, e.g., 1.23× for D1 and 1.69× for B.

Speedups from the two-level strategy were most pronounced in small systems, where much of the response vectors can be captured in the P-space. For example, a 1.87× speedup was observed for MCTDA on system A. Still, significant improvements (1.3× or more) were also achieved for large molecules like the Ni­(II) complex, despite a response space of over 600,000 parameters and a fixed P-space size of 400.

Our two-level strategy is particularly beneficial when a large number of roots is requested. For the medium-sized system C, which had the most requested roots (50), Full/Full yielded speedups of 2.05 (MCTDA) and 1.77 (MCRPA)—the largest observed in this study.

The significant speedups observed even in the absence of configuration-space response (e.g., for ROHF and CVS calculations) indicate that our two-level preconditioning strategy can be readily extended to single-reference methods such as CIS and TDDFT. These approaches require only the inactive–virtual blocks of the orbital Hessian, as listed in Tables 1 and 2, and do not involve the active Fock matrix contribution. In the case of TDDFT, an additional exchange–correlation term must be included in the orbital Hessian to account for the XC kernel contribution.^73^

## 4 Conclusions

In this work, we enhanced the efficiency of a custom Davidson algorithm for solving the CASSCF linear-response eigenvalue problem by applying a two-level strategy to both the initial eigenvector guess and the preconditioner. Inspired by analogous strategies in CI theory,^17,18^ we selected key elements of the response vectors—the so-called P space—for which full Hessian and metric matrix elements are computed and diagonalized. The remaining elements in the complementary Q space are treated using a diagonal approximation.^12–14^

In the context of MCTDA and MCRPA, P-space Hessian elements are computed for the orbital–orbital and configuration–configuration blocks, while orbital–configuration coupling blocks are neglected entirely. This decoupling enables independent initialization and preconditioning of the orbital and configuration components of the response vectors. Moreover, it allows the use of a fixed P-space size across all reference roots in state-averaged calculations.

To limit computational cost, the P-space size is restricted to a size of 400. The resolution-of-the-identity approximation is employed to reduce the prefactor of the integral transformation required for computing P-space Hessian elements. P-space orbital pairs and configurations are selected based on the lowest eigenvalues of the diagonal Hessian and metric. For the orbital response part of the diagonal Hessian, we avoid computing the most expensive two-electron integrals by employing approximations proposed by Chaban et al.^40^

We assessed the performance of our approach across a diverse set of molecular systems, varying in size, active space, and number of excited states. As expected, the two-level strategy substantially reduced both the number of Davidson iterations and the total wall time compared to the standard diagonal approximation. Applying Olsen’s method for generating trial vectors yielded no consistent additional improvement. We observed speedups ranging from 1.31 to 2.05, depending on the response vector dimension, the type of eigenvalue equation (TDA or RPA), and the number of requested roots. The most significant gains were observed for MCTDA calculations with many roots and relatively small response-vector lengths.

In future work, we plan to extend this two-level strategy to large-scale CASSCF linear-response calculations for frequency-dependent properties such as dynamic polarizabilities. More broadly, we aim to apply the same approach to other electronic structure methods, particularly time-dependent density functional theory (TDDFT) and CI singles. The two-level strategy presented here is implemented and available in ORCA 6.1.
